# Association of neuron-specific enolase values with outcomes in cardiac arrest survivors is dependent on the time of sample collection

**DOI:** 10.1186/s13054-017-1766-2

**Published:** 2017-07-08

**Authors:** Dagmar Vondrakova, Andreas Kruger, Marek Janotka, Filip Malek, Vlasta Dudkova, Petr Neuzil, Petr Ostadal

**Affiliations:** Cardiovascular Center, Na Homolce Hospital, Roentgenova 2, 15000 Prague, Czech Republic

**Keywords:** Neuron-specific enolase, Cardiac arrest, Prognosis, Mild hypothermia

## Abstract

**Background:**

Despite marked advances in intensive cardiology care, current options for outcome prediction in cardiac arrest survivors remain significantly limited. The aim of our study was, therefore, to compare the day-specific association of neuron-specific enolase (NSE) with outcomes in out-of-hospital cardiac arrest (OHCA) survivors treated with hypothermia.

**Methods:**

Eligible patients were OHCA survivors treated with targeted temperature management at 33 °C for 24 h using an endovascular device. Blood samples for NSE levels measurement were drawn on days 1, 2, 3, and 4 after hospital admission. Thirty-day neurological outcomes according to the Cerebral Performance Category (CPC) scale and 12-month mortality were evaluated as clinical end points.

**Results:**

A total of 153 cardiac arrest survivors (mean age 64.2 years) were enrolled in the present study. Using ROC analysis, optimal cutoff values of NSE for prediction of CPC 3–5 score on specific days were determined as: day 1 > 20.4 mcg/L (sensitivity 63.3%; specificity 82.1%; *P* = 0.002); day 2 > 29.0 mcg/L (72.5%; 94.4%; *P* < 0.001); and day 3 > 20.7 mcg/L (94.4%; 86.7%; *P* < 0.001). The highest predictive value, however, was observed on day 4 > 19.4 mcg/L (93.5%; 91.0%; *P* < 0.001); NSE value >50.2 mcg/L at day 4 was associated with poor outcome with 100% specificity and 42% sensitivity. Moreover, NSE levels measured on all individual days also predicted 12-month mortality (*P* < 0.001); the highest predictive value for death was observed on day 3 > 18.1 mcg/L (85.3%; 72.0%; *P* < 0.001). Significant association with prognosis was found also for changes in NSE at different time points. An NSE level on day 4 > 20.0 mcg/L, together with a change > 0.0 mcg/L from day 3 to day 4, predicted poor outcome (CPC 3–5) with 100% specificity and 73% sensitivity.

**Conclusions:**

Our results suggest that NSE levels are a useful tool for predicting 30-day neurological outcome and long-term mortality in OHCA survivors treated with targeted temperature management at 33 °C. The highest associations of NSE with outcomes were observed on day 4 and day 3 after cardiac arrest.

## Background

The incidence of out-of-hospital cardiac arrest (OHCA) in Europe and in North America has been estimated to be 50 to 100 per 100,000 of the general population [1]. Despite recent advances in cardiopulmonary resuscitation and post-resuscitation care, survival rates for sudden cardiac arrest remain poor [1–3]. Among patients who achieve return of spontaneous circulation (ROSC), reasons for poor outcome include brain injury, myocardial dysfunction and systemic ischemia-reperfusion injury [2, 4]. The severity of these three primary components of post-cardiac arrest syndrome is not uniform and depends on the cause of OHCA, the duration of the cardiac arrest and the extent of ischemic injury. Brain damage has been shown to be the most common cause of poor neurological outcome and death after cardiac arrest [5]. To date, the only therapeutic intervention supported by clinical evidence of improved neurological outcome is the management of target core temperature at 33.0 °C or 36.0 °C for 12 h to 24 h [3, 6–9]. However, even if the best evidence-based recommended therapy is provided, a substantial proportion of patients remain comatose after normalization of body temperature and withdrawal of sedation. Rapid and precise prognostication in these patients is crucial for the selection of the most appropriate diagnostic or therapeutic strategy, including possible withdrawal of further care in hopeless cases. Although prognostic options for these patients exist, they are currently limited. Recent guidelines recommend multimodal strategy for prognostication in cardiac arrest survivors. The absence of brain stem reflexes and bilateral absence of cortical somatosensory-evoked potentials are the most powerful negative prognostic factors, and must be investigated first. The second line of prognostication includes electroencephalography and imaging techniques, in addition to biomarkers of brain damage, especially the estimation of neuron-specific enolase (NSE) levels. However, the current guidelines do not recommend a specific NSE threshold to accurately predict outcome after cardiac arrest [8].

NSE is a cytoplasmic glycolytic enzyme that converts 2-phosphoglycerate to phosphoenolpyruvate. The enzyme exists as a dimer and has three distinct subunits: α, β and γ; the brain contains two α and γ subunits, but not β. The dimeric αα form is specific for glial cells, whereas γ-enolase has been shown to be located in neurons and in other cells of neuroectodermal origin. The term ‘neuron-specific enolase’ refers to both the γγ and αγ forms; these two forms of the enzyme are also present in erythrocytes and platelets. Hemolysis may, therefore, cause an increase in the serum level of NSE proportional to the degree of hemolysis, even in the absence of brain injury. The serum half-life of NSE is approximately 24 h. NSE has been widely studied as a marker for neurological prognostication. As recently as 2006, NSE levels > 33 ng/mL determined within 48 h in patients not treated with hypothermia was identified as a reliable marker for poor outcome in the large Prognosis in Postanoxic Coma (PROPAC) study [10]. This level was subsequently adopted as an American Academy of Neurology practice parameter [11]. In a recent analysis of the Targeted Temperature Management (TTM) trial [9], it was found that different core temperatures (33 °C or 36 °C) did not significantly affect NSE levels [12]. The measurement of NSE is currently recommended as an additional tool for prognostication, and high serum values at 48 h to 72 h after cardiac arrest support the prognosis of a poor neurological outcome, especially if repeated sampling demonstrates persistently high values [3, 8]. To date, however, the day-specific short-term and long-term prognostic value of NSE levels in OHCA survivors is not fully understood [13]. Accordingly, the aim of the present study was to assess the prognostic value of NSE measured daily during the first 4 days after hospital admission for OHCA.

## Methods

The present study was performed in accordance with the Declaration of Helsinki, and the study protocol was approved by the Institutional Ethics Committee of the Na Homolce Hospital (Prague, Czech Republic). Surviving patients with favorable neurological outcomes, and family members of deceased subjects or individuals with unfavorable neurological outcomes provided informed consent retrospectively. Blood samples drawn from patients who were not willing to participate in the study (expressed by family members in cases involving deceased relatives or those with unfavorable neurological outcomes) were discarded and the clinical data were not used in the analysis.

### Patients

Patients who experienced OHCA between January 2012 and March 2015, with persistent coma (Glasgow Coma Score ≤ 8) were eligible to participate in the present study. Hypothermia was initiated in the ambulance before hospital admission or at hospital admission by infusion of ice-cold saline at a rate of 30 mL/kg/h. Infusion continued until the patient’s core temperature reached 34 °C (maximum 150 min in all patients). An endovascular controlled target temperature management method (Thermogard XP, Zoll, Chelmsford, MA, USA) was started as soon as possible within 60 min of hospital admission. A target core temperature of 33 °C and a 24-hour hypothermia protocol was used in all subjects. Patients with ST-elevation myocardial infarction, and those with hemodynamic instability or repeated ventricular tachycardia or ventricular fibrillation, underwent urgent coronary angiography and percutaneous coronary intervention when applicable. Patients with extracorporeal cardiopulmonary resuscitation (ECPR) and those on intra-aortic balloon (IABP) pump were excluded from the study group.

### Blood sampling

Blood samples for the measurement of NSE levels were drawn on the first morning of hospitalization (6 to 30 h from collapse, day 1) and then every subsequent morning of hospitalization (day 2 [30 to 54 h from collapse], day 3 [54 to 78 h from collapse] and day 4 [78 to 102 h from collapse]). Serum was immediately separated by centrifugation at 1500 rpm for 5 min, and aliquots were stored at –20 °C for up to 7 days after blood withdrawal until measurement. NSE levels were then measured using an immunoradiometric assay kit (Beckman Coulter, Brea, CA, USA). Investigators performing the laboratory analyses were blinded to the clinical results; furthermore, NSE values were not available for clinical purposes and did not influence therapeutic approaches or decision-making processes.

### Clinical end points

Neurological outcome was assessed at 30 days postadmission according to the Cerebral Performance Category (CPC) scale [14]: CPC 1 – no neurological deficit; CPC 2 – mild to moderate dysfunction; CPC 3 – severe dysfunction; CPC 4 – coma; and CPC 5 – death. Physicians performing the CPC assessment were blinded to patient NSE levels. One-year mortality data were obtained from the national registry.

### Statistical analysis

Continuous variables with normal distribution are presented as mean ± SD, continuous variables with non-Gaussian distribution are shown as median (minimum [min], maximum [max]) and categorical variables are presented as percentages. Comparison of NSE distribution between CPC groups was performed with the Wilcoxon rank sum test. Receiver operating characteristic (ROC) curves were constructed to determine the sensitivity and specificity of the NSE levels at each time point (and the maximal NSE level recorded during the 4-day period) for predicting outcomes. The ROC curves for individual days were compared using the DeLong test [15]. Moreover, ROC analysis was performed also for the changes in NSE values at individual days and for each optimal cutoff value was calculated also a ratio as 100*(Dx-Dy)/Dx. NSE values at individual days and maximal NSE value were also added to a clinical multivariate logistic model containing age, gender, baseline lactate and pH, time to ROSC, initial rhythm and EEG (burst-suppression or status epilepticus). *P* < 0.05 was considered to be statistically significant. The ROC analyses were performed using MedCalc version 12 (MedCalc, Mariakerke, Belgium); all other statistical tests were performed with GraphPad Prism version 5 (GraphPad Software, La Jolla, CA, USA).

## Results

**Fig. 1:**
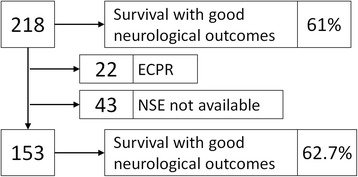
Study flow diagram. *ECPR* extracorporeal cardiopulmonary resuscitation, *NSE* neuron-specific enolase

**Fig. 2:**
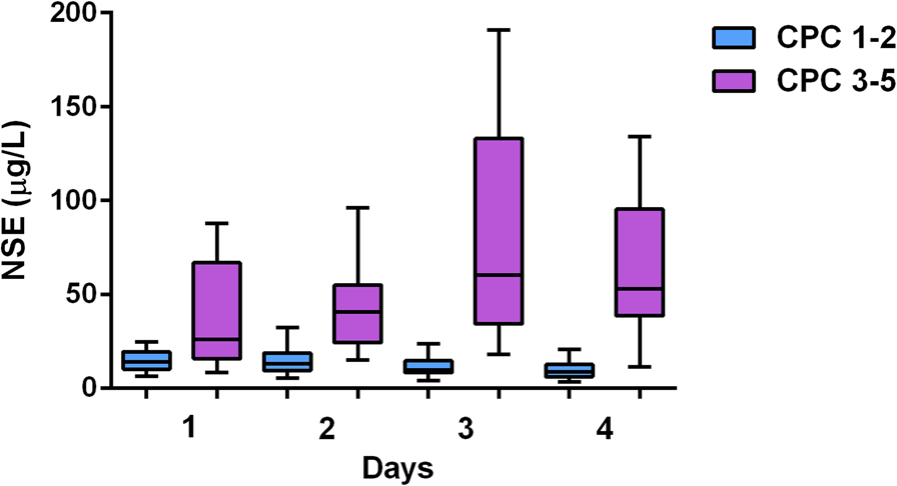
Neuron-specific enolase (NSE) levels measured on individual days. The NSE levels were significantly lower in the CPC 1–2 group in comparison with the CPC 3–5 group at each time point (*P* < 0.05). *CPC* Cerebral Performance Category

During the recruitment period, 218 OHCA survivors were admitted and hospitalized; 153 subjects were ultimately enrolled (Fig. 1). Baseline characteristics of the study group are summarized in Table 1. The median NSE level on day 1 was 16.8 mcg/L (min 6.6 mcg/L, max 196.2 mcg/L [n = 66]); on day 2 18.3 mcg/L (min 5.5 mcg/L, max 123.6 mcg/L [n = 112]); on day 3 14.6 mcg/L (min 4.1 mcg/L, max 368 mcg/L [n = 108]); and on day 4 12.0 mcg/L (min 3.5 mcg/L, max 300.0 mcg/L [n = 96]). The NSE levels were significantly lower in the CPC 1–2 group in comparison with the CPC 3–5 group at each time point (*P* < 0.05): on day 1 14.1 mcg/L versus 26.1 mcg/L; on day 2 13.1 mcg/L versus 40.8 mcg/L; on day 3 9.9 mcg/L versus 60.8 mcg/L; and on day 4 8.7 mcg/L versus 53.0 mcg/L (Fig. 2).

**Table 1:** Baseline characteristics and clinical outcomes of the study group (n = 153)

Baseline characteristic	
Age, years	64.2 ± 12.2
Male sex	115 (74.4)
Return of spontaneous circulation	21.7 ± 15.9
Blood pH at admission	7.2 ± 0.2
Lactate at admission, mmol/L	6.3 ± 4.1
VT/VF	119 (77.3)
Urgent coronary angiography	126 (82.4)
Percutaneous coronary intervention	104 (68.0)
Clinical outcomes	
30-day	
CPC 1–2	96 (62.7)
CPC 3–4	23 (15.0)
CPC 5	34 (22.2)
12-month mortality	46 (30.1)

Data presented as mean ± SD or n (%). *CPC* Cerebral Performance Category, *VF* ventricular fibrillation, *VT* ventricular tachycardia

### NSE and 30-day neurological outcomes

**Fig. 3:**
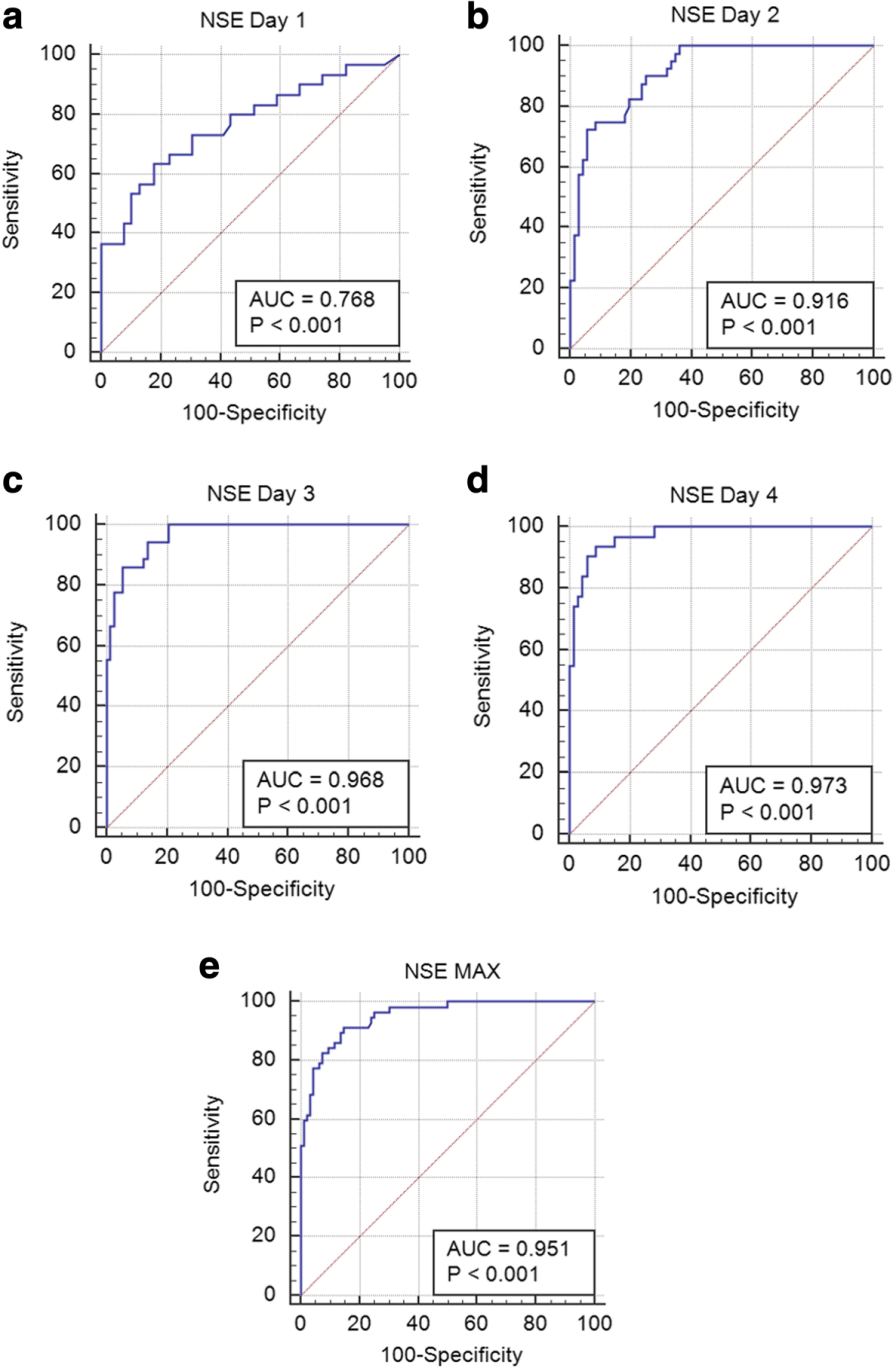
Association of neuron-specific enolase (NSE) with poor neurological outcomes (CPC 3–5). Receiver operating characteristic (ROC) curve for NSE values at Day 1 is schown on Panel **a**; ROC curve for NSE values at Day 2 is schown on Panel **b**; ROC curve for NSE values at Day 3 is schown on Panel **c**; ROC curve for NSE values at Day 4 is schown on Panel **d**; ROC curve for maximal NSE values is schown on Panel **e**. *AUC* area under the curve, *CPC* Cerebral Performance Category, *MAX* maximum

Using ROC analysis, optimal cutoff values of NSE for prediction of poor neurological outcomes (CPC 3–5) at 30 days were determined as: day 1 > 20.4 mcg/L (sensitivity 63.3%; specificity 82.1%; *P* = 0.002); day 2 > 29.0 mcg/L (sensitivity 72.5%; specificity 94.4%; *P* < 0.001); day 3 > 20.7 mcg/L (sensitivity 94.4%; specificity 86.7%; *P* < 0.001); day 4 > 19.4 mcg/L (sensitivity 93.5%; specificity 91.0%; *P* < 0.001); and maximal NSE value >27.6 mcg/L (sensitivity 91.2%; specificity 85.4%; *P* < 0.001) (Fig. 3). Comparison of ROC curves revealed that the predictive value of NSE on day 1 was significantly lower compared with other days and the maximal NSE level (Table 2). Numerically, the largest area under the curve (AUC) was found on day 4, followed by day 3 and the maximal NSE value (Fig. 3). An NSE value > 39.8 mcg/L on day 1 was associated with poor outcomes (CPC 3–5) with 100% specificity and 42% sensitivity; an NSE value > 51.1 mcg/L on day 2 was associated with poor outcomes with 100% specificity and 24% sensitivity; an NSE value > 49.2 mcg/L at day 3 was associated with poor outcomes with 100% specificity and 52% sensitivity; an NSE value > 50.2 mcg/L on day 4 was associated with poor outcomes with 100% specificity and 42% sensitivity; and maximal NSE value > 57.1 mcg/L was associated with poor outcomes with 100% specificity and 51% sensitivity. Multivariate analysis revealed that NSE at day 3 (*P* = 0.002) and day 4 (*P* = 0.007) are independent predictors of 30-day outcomes. However, not only absolute values but also differences between individual NSE levels were significantly associated with 30-day outcomes; the best predictive value for unfavorable outcome was observed with the difference between day 4 and day 1 (Table 3). Using the combination of absolute values and changes in NSE levels can markedly increase the prognostic increment of NSE measurement: an NSE level on day 4 > 20.0 mcg/L, together with a change > 0.0 mcg/L from day 3 to day 4, predicted poor outcome (CPC 3–5) with 100% specificity and 73% sensitivity.

**Table 2:** Comparison of ROC curves for day-specific neuron-specific enolase prediction of poor neurological outcome at 30 days

ROC	Difference AUC	95% Confidence interval	*P*
Day 1 – Day 2	0.277	0.074–0.480	0.0075
Day 1 – Day 3	0.273	0.072–0.474	0.0079
Day 1 – Day 4	0.296	0.095–0.498	0.0040
Day 1 – MAX	0.281	0.085–0.476	0.0049
Day 2 – Day 3	0.004	-0.016–0.023	0.6901
Day 2 – Day 4	0.020	-0.023–0.062	0.3646
Day 2 – MAX	0.004	-0.033–0.041	0.8349
Day 3 – Day 4	0.024	-0.015–0.062	0.2283
Day 3 – MAX	0.008	-0.029–0.045	0.6750
Day 4 – MAX	0.016	-0.020–0.051	0.3852

**Table 3:** ROC analysis of differences between individual values for the prediction of 30-day poor outcomes. For each optimal cutoff value was calculated also a ratio as 100*(Dx-Dy)/Dx

Difference	Optimal cutoff	Sensitivity	Specificity	AUC	*P*
D2-D1	>5.3 mcg/L (> 31%)	72.7%	90.6%	0.824	*P* < 0.001
D3-D1	> 7.1 mcg/L (> 42%)	82.4%	92.3%	0.889	*P* < 0.001
D4-D1	> 4.0 mcg/L (> 24%)	100.0%	90.9%	0.983	*P* < 0.001
D3-D2	> 2.1 mcg/L (> 11%)	89.3%	86.2%	0.878	*P* < 0.001
D4-D2	> 11.5 mcg/L (> 63%)	77.3%	95.8%	0.867	*P* < 0.001
D4-D3	> 3.8 mcg/L (> 26%)	66.7%	96.4%	0.677	*P* < 0.043

### NSE levels and 1-year mortality

**Fig. 4:**
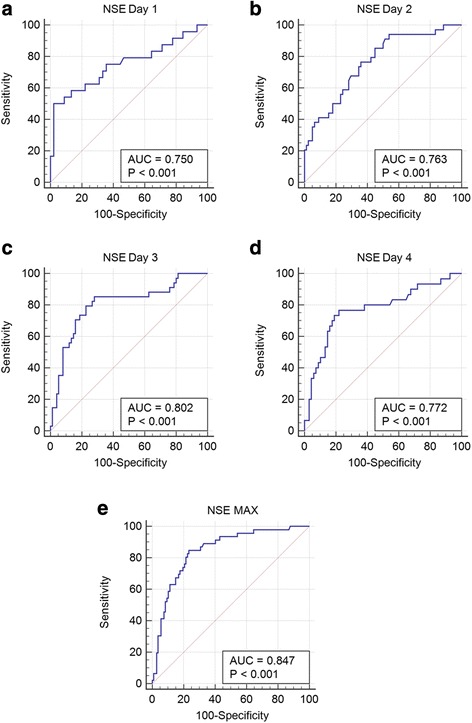
Association of neuron-specific enolase (NSE) with 1-year mortality. Receiver operating characteristic (ROC) curve for NSE values at Day 1 is schown on Panel **a**; ROC curve for NSE values at Day 2 is schown on Panel **b**; ROC curve for NSE values at Day 3 is schown on Panel **c**; ROC curve for NSE values at Day 4 is schown on Panel **d**; ROC curve for maximal NSE values is schown on Panel **e**.  *AUC* area under the curve, *MAX* maximum

ROC analyses showed that optimal cutoff values for NSE and prediction of death at 1-year follow-up were determined as: day 1 > 35.8 mcg/L (sensitivity 50.0%; specificity 97.8%; *P* < 0.001); day 2 > 18.7 mcg/L (sensitivity 76.5%; specificity 64.1%; *P* < 0.001); day 3 > 18.1 mcg/L (sensitivity 85.3%; specificity 72.0%; *P* < 0.001); day 4 > 18.5 mcg/L (sensitivity 76.7%; specificity 77.9%; *P* < 0.001); and maximal NSE value > 29.1 mcg/L (sensitivity 84.8%; specificity 76.6%; *P* < 0.001) (Fig. 4). Prediction of death within 1 year with 100% specificity was associated with very low sensitivity not exceeding 20.6%. The ROC curves were not significantly different: the numerically largest AUC was observed for maximal NSE values, followed by NSE measurements at day 3 and day 4 (Fig. 4). Multivariate analysis revealed that the maximal NSE level was an independent predictor of death. Significant association with 1-year mortality was found also for selected differences between individual NSE values (Table 4).

**Table 4:** ROC analysis of differences between individual NSE values for the prediction of 1-year mortality. For each optimal cutoff value was calculated also a ratio as 100*(Dx-Dy)/Dx

Difference	Optimal cutoff	Sensitivity	Specificity	AUC	*P*
D2-D1	> 6.8 mcg/L (> 40%)	52.6%	80.2%	0.603	*P* = 0.310
D3-D1	> 1.5 mcg/L (> 9%)	76.5%	73.1%	0.716	*P* = 0.019
D4-D1	> 7.1 mcg/L (> 42%)	73.3%	90.0%	0.730	*P* = 0,031
D3-D2	> 2.1 mcg/L (> 11%)	76.9%	78.3%	0.728	*P* = 0.001
D4-D2	> 11.5 mcg/L (> 63%)	58.3%	89.1%	0.695	*P* = 0.009
D4-D3	> 4.3 mcg/L (> 29%)	51.8%	92.7%	0.599	*P* = 0.227

## Discussion

We determined that NSE levels measured during the first 4 days after hospital admission in OHCA survivors were significantly associated with short- and long-term prognosis. However, the major result of our study was the observation that the association of NSE with outcomes significantly differed depending on the day of measurement. Furthermore, to our knowledge, we have for the first time in the larger cohort described the strong prognostic significance of NSE assessment on day 4 after hospital admission in OHCA survivors and we have found also significant association of the changes in NSE levels with clinical outcomes, especially the change from day 1 to day 4. Measurement of NSE the fourth day after cardiac arrest may be helpful particularly in patients remaining unconscious at that time.

Although the NSE levels at all individual days of measurement predicted 30-day neurological outcomes, our results indicate that association with outcomes vary depending on the time of blood sampling. The NSE levels measured on day 1 offered significantly weaker association compared with the other days and the maximal NSE level. Numerically, the strongest association with outcomes was observed for NSE level on day 4, followed by the value on day 3 and the maximal value. On the other hand, the association of individual NSE measurements with 1-year mortality did not significantly differ. Numerically, the strongest association was found for the maximal NSE value, followed by assessment on day 3 and day 4. The associations with 1-year mortality were lower compared to the associations with 30-day outcomes.

The international guidelines for post-cardiac arrest care neither recommend a specific NSE cutoff for prognosis nor an optimal time for blood sampling [8]. Current literature evidence shows a wide range of NSE cutoff values for early prognostication, frequently measured at different time points [8]. The variability of NSE values in the reported data can, in part, be explained by the high sensitivity of measurement to blood sample handling and storage conditions – hemolysis, for example, may cause a significant increase in NSE values [16, 17] – the measurement can also be influenced by the type of assay used [16–18]. Additionally, the majority of published studies have reported a limited sample size [8]. Therefore, in our study, blood samples from all patients were processed immediately after withdrawal and analyzed within 1 week to decrease the length of storage and to mitigate the risk for hemolysis and NSE degradation. Moreover, blood samples were obtained every morning of hospitalization, which although controversial from a scientific perspective, more closely reflects routine clinical practice.

Our results are consistent with previous studies reporting an association between NSE levels and prognosis in OHCA survivors treated with therapeutic hypothermia. The largest published study with serial NSE measurement was the analysis of the TTM trial [12], which included almost 700 patients. The authors found significant predictive value for NSE for 6-month outcomes at three different time points: 24 h, 48 h and 72 h after ROSC, which is concordant with our results despite the marked differences between the TTM analysis and our study regarding methods of NSE measurement or target temperature management. Of note, the AUC values for 6-month prognosis in the analysis by Stammet et al. [12] are numerically lower compared with 30-day results in our study and higher than our 1-year results. We speculate that this decreasing trend with the time from OHCA may reflect better value of NSE for short-term prognostication. Our results are consistent with several other studies reporting serial measurement of NSE and showing an association between clinical outcomes and NSE levels assessed at 24 h, 48 h, and 72 h [19–21]; at 24 h and 72 h [22]; at 24 h, 36 h, and 48 h [23, 24]; at 24 h and 48 h [25]; or at 24 h, 48 h, and 96 h [26]. None of the above-mentioned studies, however, performed statistical analysis for comparison of ROC curves on individual days. We evaluated the relation of NSE levels to short-term prognosis and to 1-year mortality. The long-term prognosis is definitely the more robust information; however, the short-term prognosis may at least help to provide correct information to patient’s next of kin.

Our study had several limitations, the first of which was a limited sample size of only 153 subjects. However, compared with other similar investigations, there were only a few with a larger cohort. The analytic method used in our study was already partly discussed above. Before analyzing samples, we did not assess the index of hemolysis; however, we mitigated the risk for hemolysis by immediate sample processing and we excluded the subjects with ECPR or IABP (ECPR and IABP are known confounders increasing hemolysis generating falsely high NSE levels). However, we cannot rule out completely that the NSE levels were affected by hemolysis. Another major limitation was the absence of values in our dataset caused by patient deaths before sampling or the unavailability of blood samples, which may have decreased the statistical power of our analysis but not the reliability of the results. Although the selection bias for the missing values is improbable it cannot be fully excluded. All patients in our study underwent mild hypothermia using endovascular body temperature control. Although our results can only be applied to patients with these characteristics, it has been shown that hypothermia probably does not influence NSE values [12, 23, 27, 28]. Despite that our results indicate marked prognostic impact of the NSE measurement, the individual value could be always influenced by method of measurement, hemolysis or other confounders; we would therefore recommend to use NSE only as an additive prognostic tool in multimodal approach in compliance with the recent European Resuscitation Council Guidelines [8].

## Conclusions

Our results suggest that NSE levels are a useful tool for predicting 30-day neurological outcome and long-term mortality in OHCA survivors treated with targeted temperature management at 33 °C. The highest associations of NSE with outcomes were observed on day 4 and day 3 after cardiac arrest.

## Abbreviations

AUC: Area under curve
CPC: Cerebral Performance Category
ECPR: Extracorporeal cardiopulmonary resuscitation
EEG: Electroencephalography
IABP: Intra-aortic balloon pump
NSE: Neuron-specific enolase
OHCA: Out-of-hospital cardiac arrest
ROC: Receiver operating characteristic
ROSC: Return of spontaneous circulation
